# *Posidonia oceanica* (L.) Delile Ethanolic Extract Modulates Cell Activities with Skin Health Applications

**DOI:** 10.3390/md16010021

**Published:** 2018-01-10

**Authors:** Laura Cornara, Giulia Pastorino, Barbara Borghesi, Annalisa Salis, Marco Clericuzio, Carla Marchetti, Gianluca Damonte, Bruno Burlando

**Affiliations:** 1 Department of Earth, Environment and Life Sciences, University of Genova, Corso Europa 26, 16132 Genova, Italy; cornara@dipteris.unige.it (L.C.); giuliapastorino@live.it (G.P.); babsonline@icloud.com (B.B.); 2 Biophysics Institute, National Research Council (CNR), via De Marini 6, 16149 Genova, Italy; carla.marchetti@ge.ibf.cnr.it; 3 Center of Excellence for Biomedical Research (CEBR), University of Genova, Viale Benedetto XV 5, 16132 Genova, Italy; annalisa.salis@unige.it; 4 Department of Sciences and Technological Innovation, University of Eastern Piedmont, Viale T. Michel 11, 15121 Alessandria, Italy; marco.clericuzio@uniupo.it; 5 Department of Experimental Medicine, Section of Biochemistry, University of Genova, Viale Benedetto XV 1, 16132 Genova, Italy; Gianluca.Damonte@unige.it; 6 Department of Pharmacy, University of Genova, Viale Benedetto XV 3, 16132 Genova, Italy

**Keywords:** adipocytes, chicoric acid, collagen type I, fibroblasts, lipolysis, melanin, tyrosinase

## Abstract

Seagrasses are high plants sharing adaptive metabolic features with both terrestrial plants and marine algae, resulting in a phytocomplex possibly endowed with interesting biological properties. The aim of this study is to evaluate the in vitro activities on skin cells of an ethanolic extract obtained from the leaves of *Posidonia oceanica* (L.) Delile, family Potamogetonaceae, herein named *Posidonia* ethanolic extract (PEE). PEE showed high radical scavenging activity, high phenolic content, and resulted rich in chicoric acid, as determined through HPLC-MS analysis. The use of MTT assay on fibroblasts showed a PEE cytotoxicity threshold (IC_05_) of 50 µg/mL at 48 h, while a sub-toxic dose of 20 µg/mL induced a significant increase of fibroblast growth rate after 10 days. In addition, an ELISA assay revealed that PEE doses of 5 and 10 µg/mL induced collagen production in fibroblasts. PEE induced dose-dependent mushroom tyrosinase inhibition, up to about 45% inhibition at 1000 µg/mL, while 50% reduction of melanin was observed in melanoma cells exposed to 50 µg/mL PEE. Finally, PEE lipolytic activity was assessed by measuring glycerol release from adipocytes following triglyceride degradation. In conclusion, we have collected new data about the biological activities of the phytocomplex of *P. oceanica* seagrass on skin cells. Our findings indicate that PEE could be profitably used in the development of products for skin aging, undesired hyperpigmentation, and cellulite.

## 1. Introduction

Over the last years, the herbal market has rapidly increased and many surveys have been conducted that aim at finding natural ingredients with possible applications as food additives or medicine. In this area, special attention has been given to the sea environment as a rich source of new active compounds [1]. Among marine botanical organisms, algae have been deeply investigated and exploited [2]; in contrast, the phytopharmaceutical profile of seagrasses is still almost unknown. However, these latter are high plants sharing adaptive metabolic features with both terrestrial plants and seaweed, resulting in a peculiar phytocomplex possibly endowed with interesting biological properties [3].

*Posidonia oceanica* (L.) Delile, family Potamogetonaceae, is a long-living, slow-growing, endemic Mediterranean seagrass forming extensive meadows in coastal shallow waters [4]. The plant undergoes massive leaf loss in autumn, giving rise in some areas to conspicuous beach deposits. The profile of secondary metabolites is mostly characterized by phenolic compounds, as also testified by the presence of so-called tannin cells [5]. Chicoric acid is generally reported as the major constituent of leaves [6,7], caftaric and gentisic acids are also abundant in leaves, and other phenolic constituents include the aldehyde vanillin, and *p*-coumaric, ferulic, caffeic, and cinnamic acids [8]. Other chemical constituents isolated from the plant include phenol derivatives (e.g., phloroglucinol), benzoic acid derivatives (e.g., *p*-hydroxybenzoic and vanillic acids), calchones, and proanthocyanidins [9]. Lignin is present in all tissues [10], while major flavonoids include the flavonols kaempferol, quercetin, isorhamnetin (about 1–3 μg g^−1^ dw), and myricetin (about 20 μg g^−1^ dw) [11]. The lipid fraction has been found to contain prevalently palmitic, palmitoleic, oleic, and linoleic acids, in addition to the phytosteroids campesterol, stigmasterol, and β-sitosterol [12]. A novel sesquiterpenic alcohol named posidozinol has also been isolated from the leaves [13].

The plant exploitation has mostly concerned beached leaf litter, used in the past as packing material for glassware and pottery, roof insulation, shipping of fishery products, cattle bedding, mattress and pillow filling, and still used in some cases as cattle forage and compost [14]. News on medicinal uses date back to ancient Egypt, where it was supposedly used for skin disease [15], while more recently in the southwestern Mediterranean region the plant has been used as a remedy for acne, leg pain, diabetes, respiratory infections, hypertension, and colitis [16]. 

Very little scientific data on *P. oceanica* bioactivities and therapeutic properties are available. Antibacterial and antimycotic activities have been found in a rhizome extract [17], while antidiabetic and vasoprotective effects on alloxan diabetic rats have been observed upon treatment with an ethyl acetate fraction from the aqueous residue of a hydroethanolic leaf extract [18].

We obtained a *P. oceanica* ethanolic leaf extract (PEE) characterized by a high concentration of chicoric acid, as determined through HPLC-MS analysis. PEE was subjected to in vitro cell-free and cell-based tests, aimed at showing possible applications for skin care and disease. These experiments revealed stimulation of fibroblast proliferation and collagen production, anti-melanogenic activities, and the stimulation of lipolysis in adipocytes, suggesting that PEE could find applications in skin anti-aging, anti-cellulite and depigmenting products.

## 2. Results

### 2.1. Chemical Characterizations

**Figure 1:**
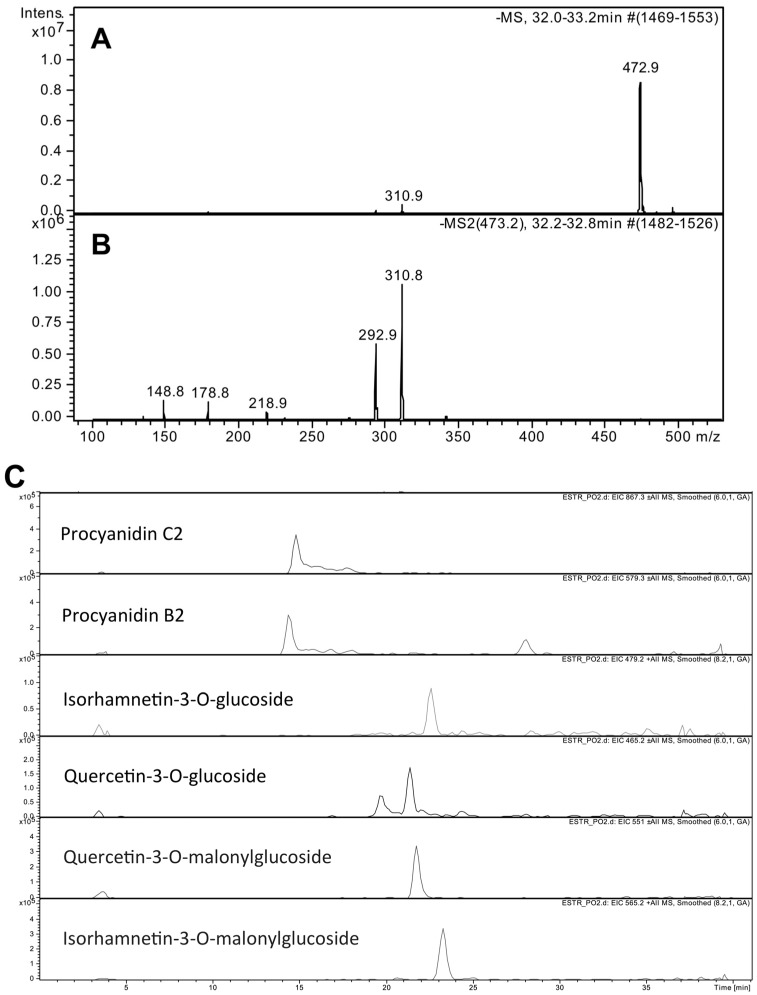
(**A**) Full scan acquired in negative ion mode and (**B**) tandem mass spectra relative to the peak at retention time (RT) = 32.5 min, in the HPLC-MS total ion chromatogram of *Posidonia* Ethanolic Extract (PEE). The molecular mass and fragment ions present are consistent with those expected for chicoric acid. (**C**) Extracted ion chromatograms acquired in positive ion mode, relative to other major PEE constituents identified by MS/MS spectra.

The PEE extract showed an IC_50_ value of 32 ± 2 μg/mL in the DPPH radical scavenging assay, corresponding to 6.5 mM (1.14 mg/mL) ascorbic acid equivalents (AAE). The extract was found to contain 126 ± 3 μg g^−1^ dw total polyphenols, expressed as gallic acid equivalents (GAE), and total iodine at 60 ± 10 μg g^−1^ dw, according to ICP-MS quantification. The HPLC-MS analysis of PEE showed, at a retention time (RT) of 32.5 min (66% solvent B), a major chromatographic peak whose full scan and fragmentation mass spectra were consistent with those expected for chicoric acid (Figure 1A,B). Quantification of the compound was achieved using a calibration curve obtained by injecting chicoric acid standard in concentrations ranging from 5 to 25 μM (linearity *r*^2^ = 0.999), yielding a value of 55.8 ± 7 mg g^−1^ dw of chicoric acid in PEE. Other major PEE compounds identified by HPLC-MS and tandem MS/MS were flavonoid molecules including procyanidin C2, procyanidin B2, isorhamnetin-3-*O*-glucoside, quercetin-3-*O*-glucoside, quercetin-3-*O*-malonylglucoside, and isorhamnetin-3-*O*-malonylglucoside (Figure 1C).

### 2.2. Fibroblast Activation

**Figure 2:**
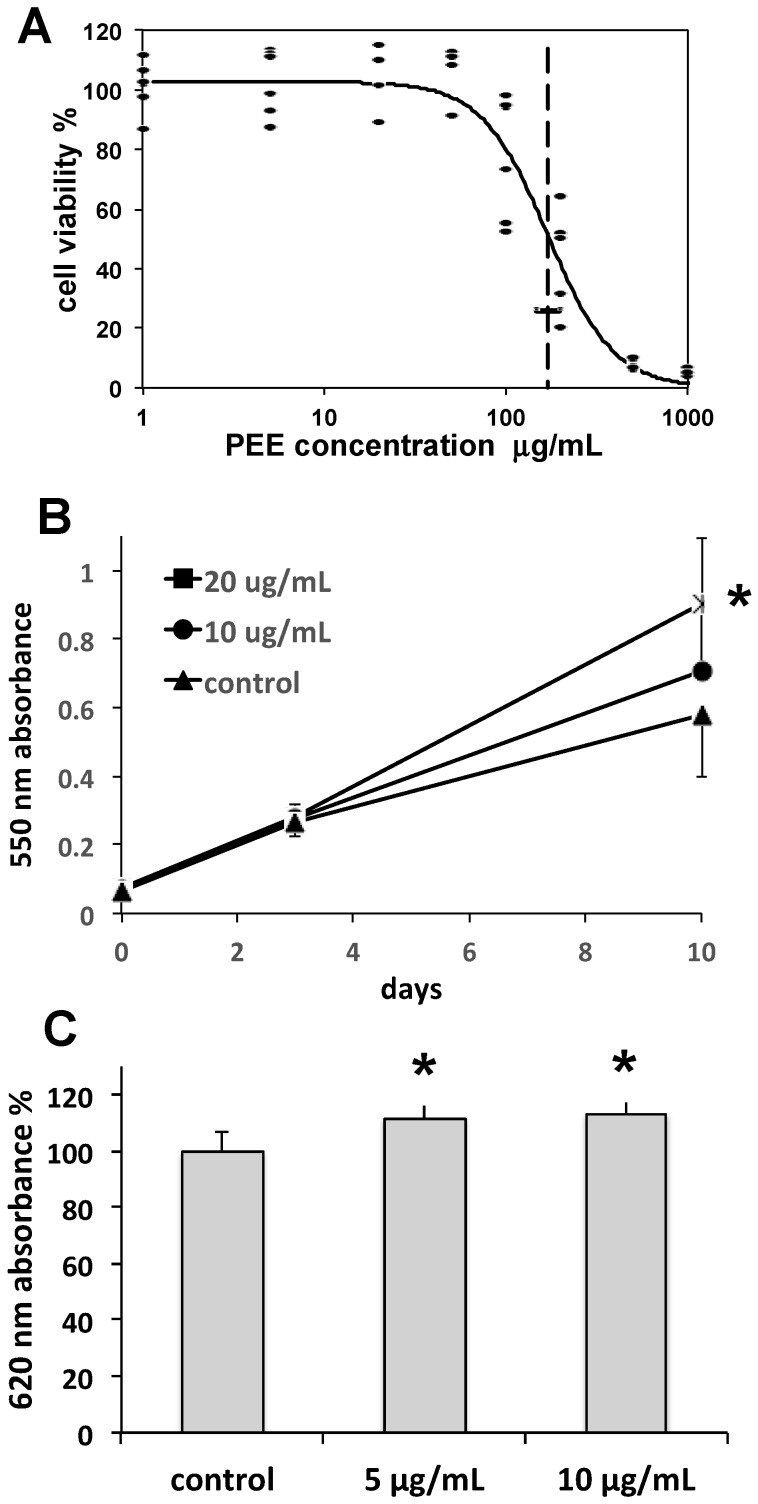
(**A**) Dose-response curve of the effect of PEE on fibroblast cell viability. Data are percent cell viabilities recorded in *n* = 6 replicates of MTT assay after 48-h incubations with different PEE concentrations. Downhill logistic best fit (continuous line), IC_50_ value (dashed vertical line), and its 95% CI (horizontal bar) are shown. (**B**) Fibroblast growth rate curves derived from the MTT assay and expressed in terms of cell densities. Data are mean 550 nm absorbances ± SD from six replicates in two independent experiments. * = *p* < 0.01 with respect to other groups at the same endpoint. (**C**) Induction of fibroblast collagen production determined by an ELISA assay after 48-h incubations with 5 and 10 µg/mL PEE. Data are mean 620 nm absorbances ± SEM from six replicates in two independent experiments. * = *p* < 0.01 with respect to the control.

Dose-response curves obtained by MTT data were analyzed by a logistic regression model as previously reported [19], allowing the estimation of the PEE effect on fibroblast viability at 48 h, with a median inhibitory concentration of IC_50_ = 170 µg/mL (95% CI = 146–197), and a toxicity threshold of IC_05_ = 50 µg/mL (95% CI = 33–73) (Figure 2A).

MTT data concerning fibroblast cell growth showed a dose-dependent increase of growth rate in fibroblasts exposed for up to 10 days to sub-toxic 20 µg/mL PEE (Figure 2B). Moreover, a significant increase of collagen production was observed in fibroblasts exposed to 5 and 10 µg/mL PEE, with respect to cells maintained under control conditions (Figure 2C).

### 2.3. Demelanizing Activity

**Figure 3:**
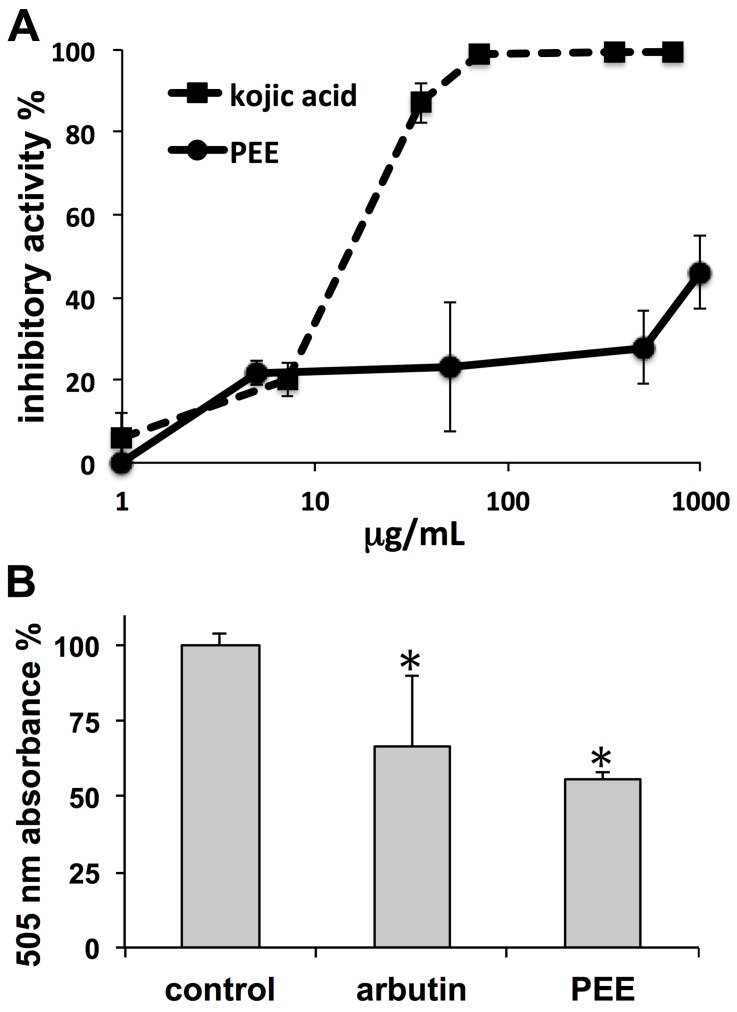
(**A**) Dose-dependent curves of mushroom tyrosinase inhibition exerted by kojic acid as a positive control (dashed line) and by PEE (continuous line). Tyrosinase activity was determined in a cell-free, in vitro assay using l-tyrosine as a substrate. Data are mean percent inhibitions ± SD from six replicates in two independent experiments (see Materials and Methods). (**B**) Inhibition of melanin production in MeWo melanoma cells after 72 h incubation with 1 mg/mL arbutin, or with 50 µg/mL PEE. Cell melanin production was quantified as mean 505 nm absorbances ± SD from three replicates in two independent experiments. * = *p* < 0.01 with respect to the control.

PEE modulatory effects on skin melanization were evaluated in free solution on mushroom tyrosinase using l-tyrosine as a substrate, as well as on MeWo melanoma cells growing in vitro. The positive control kojic acid induced dose-dependent inhibition of tyrosinase activity with an estimated IC_50_ of 14.7 µg/mL, or 2.06 µM (Figure 3A). Incubation with PEE also induced dose-dependent tyrosinase inhibition, starting from about 20% inhibition at 5 µg/mL up to about 45% inhibition at 1000 µg/mL (Figure 3A). For all PEE concentrations, the use of the *t*-test with Bonferroni correction yielded *p* < 0.01 (*n* = 6) with respect to the control (no inhibition).

Data obtained with mushroom tyrosinase were confirmed by experiments on melanoma cells, showing an about 50% reduction of melanin content induced by 50 µg/mL PEE at 72 h. Such a depigmenting effect was not statistically different (*p* > 0.05) from that obtained with the positive control arbutin (1 mg/mL) (Figure 3B). MTT analysis of PEE cytotoxicity on these cells revealed a toxicity threshold of IC_05_ > 100 µg/mL at 72 h, showing that the above melanin reduction was not due to aspecific injurious effects.

### 2.4. Lipolysis Activation

**Figure 4:**
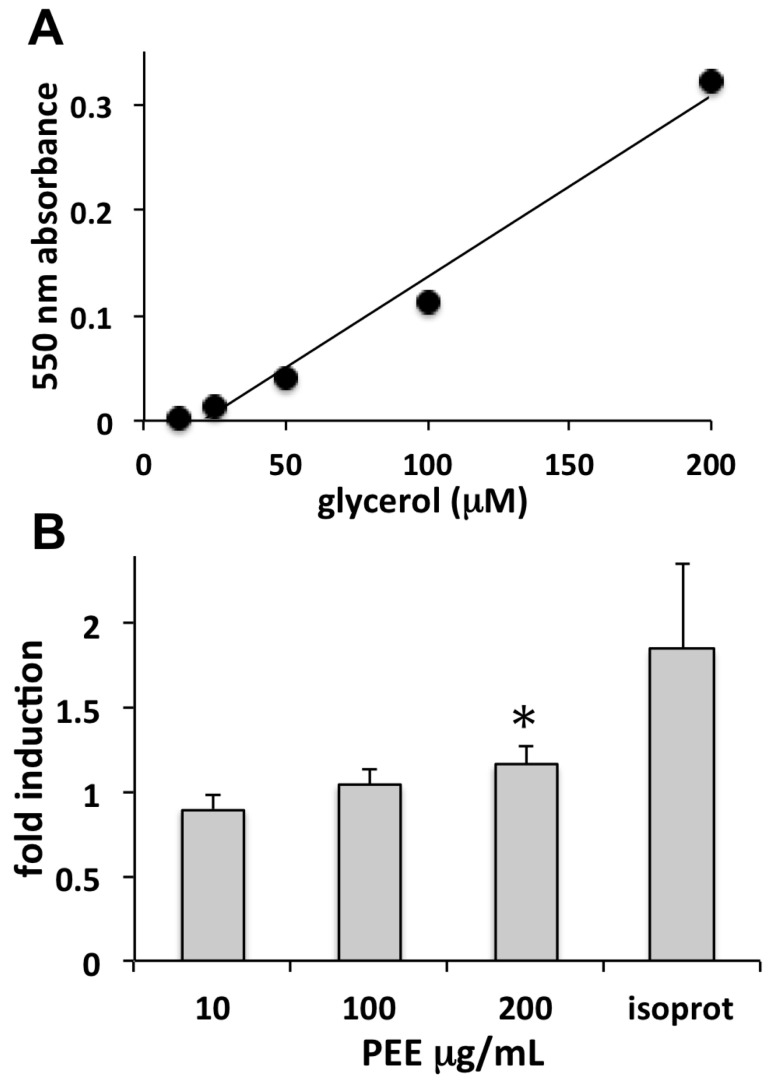
Induction of lipolysis in human adipocytes evaluated by glycerol release. (**A**) Standard curve of glycerol determined by the ZenBio Kit Human Adipocyte Lipolysis Assay Kit (see Materials and Methods). (**B**) Assay of glycerol released from adipocytes determined as above after exposure for 3 h to different concentrations of PEE, or to 1 µM isoproterenol (isoprot). Data are mean fold inductions of lipolysis ± SD, calculated as the ratio between µmoles/L of glycerol released by treated cells and by controls, obtained from three replicates in two independent experiments (* = *p* < 0.05).

PEE lipolytic activity was assessed in vitro by measuring glycerol released by adipocytes following triglyceride degradation. Determination of a standard curve for glycerol (Figure 4A) allowed for the quantification of the induction of lipolysis by PEE in terms of glycerol released by cells (Figure 4B). Data showed a dose-dependent increase of lipolysis in the range of 10–200 µg/mL PEE, reaching a significant induction at the highest concentration, though lower than that induced by the positive control isoproterenol. The MTT analysis of PEE cytotoxicity on adipocytes revealed a toxicity threshold of IC_05_ > 200 µg/mL at 48 h, showing that the observed lipolytic effect was not due to cell damage.

## 3. Discussion

This is the first study concerning the effects of a *P. oceanica* extract on skin cells, and one of the very few scientific reports about the biological properties of the *P. oceanica* phytocomplex. In agreement with literature reports about this plant, our HPLC-MS characterization showed that the major compound of PEE was chicoric acid. The resultant extract was enriched in chicoric acid, with amounts 4–5-fold higher than those reported for *P. oceanica* leaves [7]. The quantification of PEE radical scavenging activity was remarkably high among plant extracts [20], consistent with the richness in chicoric acid and other phenolic compounds typical of *P. oceanica*. Our analyses also confirmed the chemical affinity of seagrass with seaweed, since the iodine content of PEE rated within the range reported for seaweed [21]. Considering the usual doses of extracts from natural sources used in products for humans (e.g., 0.1–1%), the PEE iodine content is quite compatible with a maximum tolerable daily intake of 1.0 mg iodine set by the World Health Organization [22].

Collagen is the main constituent of the dermal matrix, while collagen type I is the most abundant isoform, forming collagen bundles. Collagen is produced by fibroblasts, is essential for skin tone and turgor, and undergoes physiological turnover through continuous degradation by matrix metalloproteinases and replacement by fibroblast neosynthesis. During skin aging, collagen degradation tends to overwhelm renewal, resulting in the formation of fine lines, wrinkles, and other alterations. Hence, the maintenance of fibroblast function is a prerequisite for contrasting skin aging. In our study, the complex of effects induced by PEE on fibroblast growth rate and collagen synthesis indicate a positive stimulation of fibroblast activity, suggesting the possible use of PEE in anti-wrinkle and anti-aging skin care formulations.

The observed PEE inhibitory activities on both tyrosinase and melanoma pigmentation indicate skin whitening properties. In tyrosinase activity assays, the strength of PEE inhibition was much lower than that of the reference compound kojic acid. Even though a different inhibition profile could be possible using l-dopa instead of l-tyrosine as a substrate, the dose-dependent curve of kojic acid fits a logistic trend, consistent with the effect of a single agent, whereas that of PEE diverges from this model, suggesting that the dose-dependent tyrosinase inhibition of PEE could be the result of multiple agents with different inhibitory patterns. In tests on melanoma cells, the PEE demelanizing effect was stronger than that of the reference compound arbutin, confirming that the PEE whitening effect could occur through a complex mechanism, with a component involving tyrosinase inhibition, and another one targeting pathways that regulate cell melanization such as MC1R-MITF [23]. However, regardless of the mechanism, the data indicate the possible use of PEE in formulations aimed at contrasting hyperpigmentation conditions, such as melasma associated with age, freckling, age spots, post-inflammatory melanization, and sites of actinic damage.

Cellulite is a skin condition associated with hypodermal fat accumulation, commonly occurring as lumps and dimples on women’s thighs. Adipocytes are fat storage cells of adipose tissue that may undergo excessive fat load, leading to the appearance of cellulite. The lipolytic induction of PEE observed in our study is indicative of a possible use in topically applied products aimed at reducing fat accumulation in adipocytes and unaesthetic cellulite.

Among the compounds of PEE that could be responsible for the observed effects, chicoric acid, the most abundant acid found in PEE, has been shown to possess antioxidant, antiangiogenic, anti-inflammatory, antiallergic, antidiabetic, and anti-HIV activities [24,25,26,27]. Among these, only the strong antioxidant power of chicoric acid can be matched to our data, whereas the other findings from our experiments are completely new for *P. oceanica*, and have not been reported for chicoric acid so far. Due to its abundance in the plant and in PEE, the complex consisting of chicoric acid and flavonoids is a major candidate for at least part of the observed PEE effects on skin cells and activities.

In conclusion, we have collected data concerning the biological effects on skin cells of a chicoric acid-rich, ethanolic extract of the seagrass *P. oceanica*. The obtained results represent a novel discovery among the studies on the biological effects of plant phytocomplexes, since they concern a seagrass that is poorly investigated from this point of view. Our findings indicate that the *P. oceanica* phytocomplex can be profitably used in the development of products for contrasting wrinkle formation and skin aging, undesired hyperpigmentation, and cellulite.

## 4. Materials and Methods

### 4.1. Reagents and Plant Material

Cell culture reagents and other chemicals were from Sigma-Aldrich (St. Louis, MO, USA), unless otherwise indicated. Fresh, beached residues of *Posidonia oceanica* (L.) Delile seagrass were collected in autumn 2015 at Favignana Island, Sicily, under the supervision of the “Area Marina Protetta Isole Egadi” natural reserve (Favignana, Italy). Plants were determined by one of the authors (LC) and voucher specimens were deposited at the Herbarium of DISTAV, University of Genova, Genova, Italy (GE s.n.).

### 4.2. Extraction

Soon after collection, leaves were separated from shoots, cleaned manually of basal sheath and epiphytes and rinsed in seawater, dehydrated for 36 h in a forced-ventilation oven at 42 °C, and grounded to a particle size of about 1–2 mm. The pulverized material (100 g) was put in a beaker, extracted under shaking in 60% aq. ethanol (1 L), and acidified with formic acid (pH 4.0) at room temperature for 4 h. The residual of the first extraction was separated from the supernatant and subjected to a second extraction as above. The supernatants of the two extraction steps were mixed together, cloth filtered, and then filtered through a Duran® sintered glass filter disc (DURAN Group GmbH, Wertheim, Germany) and vacuum-dried in a Buchi Rotavapor R-114 (Buchi Italia s.r.l., Cornaredo, Italy) under controlled temperature (<45 °C). The dried *P. oceanica* ethanolic extract (PEE) was finely pulverized with a mortar and stored at −20 °C until use. The total extraction yield was about 10% (dw/dw).

### 4.3. Radical Scavenging and Total Polyphenol Assays

Radical scavenging activity of PEE was quantified by the DPPH (2,2-diphenyl-1-picrylhydrazyl) assay, following a well-established protocol [20]. We used an EtOH solution of DPPH 10^−4^ M; a 4:1 DPPH: sample ratio, a reaction time of 30 min, and the absorbance was recorded at 516 nm. After linear regression, the antioxidant activity was calculated as IC_50_, i.e., the concentration needed to reduce the initial DPPH absorbance value to half. The above data were also compared to those recorded for a standard solution of ascorbic acid, in order to express the antioxidant power of extracts as ascorbic acid equivalents (AAE) [28].

The total polyphenol content of PEE was determined by the Folin-Ciocalteu method as previously reported [29]. A standard curve was obtained from gallic acid samples and total polyphenols in PEE were expressed as mg GAE per g of dry extract.

### 4.4. ICP-MS

Total iodine determination in PEE was performed by inductively coupled plasma-mass spectrometry, using a Thermo Scientific X Series 2 ICP-MS equipped with a perfluoroalkoxy (PFA) micro-flow concentric nebulizer (Thermo Fisher Scientific, Waltham, MA, USA). The inlet system included a PC3 Peltier chiller (Elemental Scientific, Omaha, NE, USA) and a cyclonic spray chamber. Plasma worked at the power of 1400 W; coolant, auxiliary, and nebulizer flows were set at 14.0, 0.82, and 0.96 L/min, respectively. The peristaltic pump ran at 30%, except for uptake and washout of the sample, set as fast pump (100%). The Collision Cell Technology-Kinetic Energy Discrimination (CCT-KED) mode used an H_2_/He 8/92 mixture, set at a flow of 5.0 mL/min.

An aliquot of 10 mg of PEE was weighed and put in a glass test tube with 0.15 mL of 69% HNO_3_, 1.0 mL of 30% H_2_O_2_, and 3.0 mL of ultrapure H_2_O. The test tube was heated at 90 °C in a quartz sand bath for 2 h, and then the volume of the digested solution was increased to 5.0 mL with ultrapure water. Quantitative analysis was performed by means of calibration standard solutions in the range of 1–200 μg/L. 

### 4.5. HPLC-MS

High-performance liquid chromatography coupled with mass spectrometry (HPLC-MS/MS) was performed using an Agilent 1100 HPLC-MSD Ion Trap XCT system, equipped with an electrospray ion source (HPLC-ESI-MS) (Agilent Technologies, Santa Clara, CA, USA). Separation of PEE was performed on a Symmetry C18 column 1 mm × 150 mm with 3 μm particle size (Waters Corporation, Milford, MA, USA). Eluents used were water (eluent A) and MeOH (eluent B), both added with 0.1% formic acid. The gradient employed was: 5% eluent B for 5 min, linear to 40% eluent B in 35 min, then linear gradient to 95% in 15 min, and finally hold at 95% eluent B for another 5 min. The flow rate was set to 30 µL/min and the column temperature was set to 30 °C. The injection volume was 8 μL. Ions were detected in the positive and negative ion mode, in the 200–1000 *m*/*z* range, and ion charged control with a target ion value of 200,000 and an accumulation time of 300 ms. A capillary voltage of 3300 V, nebulizer pressure of 15 psi, drying gas of 8 L/min, dry temperature of 325 °C, and 2 rolling averages (averages: 5) were the parameters set for the MS detection. MS/MS analysis was conducted using an amplitude optimized time by time for each compound.

### 4.6. Cell Culture

Stabilized human dermal fibroblasts (46BR.1N, Sigma-Aldrich, St. Louis, MO, USA) were used for cell proliferation and collagen production assays. Fibroblasts were grown in DMEM, supplemented with 10% fetal bovine serum (FBS) at 37 °C, in a 5% CO_2_, fully humidified atmosphere. 

Cells of the MeWo human melanoma cell line (ATCC, HTB-65) were used for melanin assays. These cells were cultured in RPMI supplemented with 10% FBS, 1% glutamine, and 1% antibiotic mix, at 37 °C in humidified 5% CO_2_.

Subcutaneous human preadipocytes (Zen-Bio Inc., Research Triangle Park, NC, USA) were used for lipolysis assays. Preadipocytes were grown in Preadipocyte Medium (cat# PM-1, Zen-Bio Inc.) according to the manufacturer’s protocol, at 37 °C in humidified 5% CO_2_.

### 4.7. Cell Viability and Proliferation Assays

PEE effects on cell viability were determined on 46BR.1N fibroblasts, MeWo cells, and adipocytes by the MTT assay, as previously reported [30]. Cells were settled in 96-well plates for 24 h, 10,000 cells per well, and then exposed for 48 h to a logarithmic series of PEE concentrations in DMEM, ranging between 1 and 1000 µg/mL. After the MTT reaction, plates were read at 550 nm in a VMax microplate reader (Molecular Devices, Sunnyvale, CA, USA).

Cell growth rates were determined on 46BR.1N fibroblasts by settling cells in 96-well plates at a density of 5000 cells per well, and then exposing them for periods of 3 and 10 days to 10 or 20 µg/mL PEE. At the end of exposures, an MTT assay was carried out as above, obtaining cell growth curves.

### 4.8. Collagen Production

The production of collagen type I by 46BR.1N fibroblasts was evaluated by ELISA as previously reported [30]. Briefly, cells were settled in 96-well plates and incubated with 5, 10, or 20 µg/mL PEE for 48 h. Thereafter, cells were fixed with 3.7% paraformaldehyde, blocked with BSA, probed with mouse anti-human collagen type I (ab6308, Abcam, Cambridge, UK), and then with HRP-conjugated rabbit anti-mouse IgG (ab97046, Abcam), incubated with Pierce 1Step™ Ultra TMB ELISA Substrate Solution (Thermo Fisher Scientific), blocked with 2 M sulfuric acid, and read at 620 nm in the microplate reader.

### 4.9. Cell-Free Tyrosinase Inhibition Assay

Depigmenting properties of PEE were assessed by the use of an in vitro mushroom tyrosinase inhibition assay [31]. Aliquots of 10 µL of a solution composed of 125 U/mL mushroom tyrosinase in phosphate buffer (pH 6.8) were added to 96-well plates, followed by 70 µL of phosphate buffer and 60 µL of ultrapure water, or PEE dissolved in ultrapure water, in order to obtain a series of final concentrations ranging between 5 and 1000 µg/mL of PEE. Kojic acid was used instead of PEE as a positive control. Thereafter, 70 µL of 0.3 mg/mL l-tyrosine in ultrapure water was added. Blanks without enzyme were also included for all conditions. Plates were then incubated at 30 °C for 30 min and absorbance was read at 505 nm in the microplate reader. Percent inhibitory activity (*I*%) was calculated according to the formula:

(1) $$I\%=\left(1-\frac{\left(A_{\mathrm{en}/\mathrm{ex}}-A_{\mathrm{ex}}\right)}{\left(A_{\mathrm{en}}-A_{\mathrm{bk}}\right)}\right)\times 100,$$

where *A*_ex/en_ = absorbance of assay mixture with extract and enzyme; *A*_ex_ = absorbance of assay mixture with extract and without enzyme; *A*_en_ = absorbance of assay mixture with enzyme and without extract; *A*_bk_ = absorbance of assay mixture without enzyme and extract (blank).

### 4.10. Melanin Assay

Confluent MeWo cells were suspended and plated in 24-well plates (100,000 cells/well), allowed to settle for 24 h, and then exposed to PEE at a dose of 50 µg/mL for 72 h. Treatment with arbutin (1 mg/mL) was used as a positive control. Thereafter, the culture medium was removed, cells were washed with PBS, trypsinized, centrifuged, and the pellet was subjected to freeze-thawing. The pellet was then dissolved in 100 µL of 1 N NaOH and the crude extract was assayed with the microplate reader at 505 nm to determine the melanin content. All tests were performed in triplicate.

### 4.11. Lipolysis Assay

The lipolytic effect of PEE was evaluated on primary human adipocytes using the ZenBio Cellulite Treatment Screening Kit Human Adipocyte Lipolysis Assay Kit (cat# LIP-1-L1; LIP-1-NCL1, Zen-Bio Inc., Research Triangle Park, NC, USA) for the detection of free glycerol. Pre-adipocytes were grown as above, settled in 96-well plates, differentiated into adipocytes for one week in Adipocyte Differentiation Medium (cat# DM-2), and maintained for a further week in Adipocyte Medium (cat# AM-1). Fully differentiated adipocytes were incubated for 3 h with 10, 100, or 200 µg/mL PEE, and samples of conditioned medium were then assayed for glycerol as previously reported [29]. Samples were read in the microplate reader at 550 nm. Absorbance increase is proportional to glycerol concentration in the sample.

### 4.12. Statistics

Data were analyzed with the R package, version 3.0.1 (http://www.r-project.org/foundation/), using Student’s *t*-test with Bonferroni’s correction for multiple comparisons. The difference between two conditions was considered significant if *p* < 0.05.
